# Prevention of Diabetes-Associated Cognitive Dysfunction Through Oral Administration of Lipopolysaccharide Derived From *Pantoea agglomerans*

**DOI:** 10.3389/fimmu.2021.650176

**Published:** 2021-08-27

**Authors:** Haruka Mizobuchi, Kazushi Yamamoto, Masashi Yamashita, Yoko Nakata, Hiroyuki Inagawa, Chie Kohchi, Gen-Ichiro Soma

**Affiliations:** ^1^Control of Innate Immunity, Collaborative Innovation Partnership, Kagawa, Japan; ^2^Research and Development Department Macrophi Inc., Kagawa, Japan; ^3^Research Institute for Healthy Living, Niigata University of Pharmacy and Applied Life Sciences, Niigata, Japan

**Keywords:** microglia, lipopolysaccharide, oral administration, neuroprotection, cognitive dysfunction, dementia

## Abstract

Diabetes-related cognitive dysfunction (DRCD) is a serious complication induced by diabetes. However, there are currently no specific remedies for DRCD. Here, we show that streptozotocin-induced DRCD can be prevented without causing side effects through oral administration of lipopolysaccharide (LPS) derived from *Pantoea agglomerans*. Oral administration of LPS (OAL) prevented the cerebral cortex atrophy and tau phosphorylation induced by DRCD. Moreover, we observed that neuroprotective transformation of microglia (brain tissue-resident macrophages) is important for preventing DRCD through OAL. These findings are contrary to the general recognition of LPS as an inflammatory agent when injected systemically. Furthermore, our results strongly suggest that OAL promotes membrane-bound colony stimulating factor 1 (CSF1) expression on peripheral leukocytes, which activates the CSF1 receptor on microglia, leading to their transformation to the neuroprotective phenotype. Taken together, the present study indicates that controlling innate immune modulation through the simple and safe strategy of OAL can be an innovative prophylaxis for intractable neurological diseases such as DRCD. In a sense, for modern people living in an LPS-depleted environment, OAL is like a time machine that returns microglia to the good old LPS-abundant era.

## Introduction

Diabetes affects 463 million people worldwide, and one of its more serious complications is diabetes-related cognitive dysfunction (DRCD) (1). However, there is still no radical preventive or curative treatment for DRCD. Accordingly, its prevention is a crucial to sustained global health.

To address this problem, we focused on oral administration of lipopolysaccharide (LPS) derived from *Pantoea agglomerans*. Our previous study showed that oral administration of LPS derived from *P. agglomerans* (OAL) prevents high fat diet-induced cognitive dysfunction (2). In addition, *P. agglomerans* is reportedly used as a food preservative (3, 4), and the safety of OAL is objectively guaranteed based on Organization for Economic Co-operation and Development standards (5–7). Based on these findings, we hypothesized that OAL could prevent DRCD.

Here, we demonstrate the effect of OAL on preventing DRCD using an intracerebroventricular injection model of the diabetes inducer, streptozotocin (STZ), which has already been established as a DRCD model (8–10). Furthermore, in order to elucidate the DRCD prevention mechanism utilized by OAL, we depleted microglia, which are brain tissue macrophages, and performed genetic analysis of isolated microglia. The results revealed that transformation to neuroprotective microglia is required for DRCD prevention by OAL. Importantly, it is strongly suggested that signal activation of colony stimulating factor 1 receptor (CSF1R) by the membrane-bound form CSF1 is involved in this transformation to neuroprotective microglia.

## Materials And Methods

### Animals and Tissue Sample Preparation

Six-week-old male C57BL/6 mice (20–22 g) were purchased from SLC, Inc., Shizuoka, Japan, and were acclimated for 1 week. All mice (3–5 mice per cage) were maintained under specific pathogen-free conditions in a temperature- and humidity-controlled room under a 12-h light/dark cycle with unrestricted access to food and water. Mouse diet D12450B was purchased from Research Diets, Inc., New Brunswick, NJ, USA. At the end of the experiments, mice were anaesthetized under 4% isoflurane vapor using the simple inhalation anesthesia device NARCOBIT-E (Natsume Seisakusho Co., Ltd., Tokyo, Japan) and euthanized by cardiac puncture whole blood collection. The animal experiments were reviewed and approved by the Animal Care and Use Committee of the Control of Innate Immunity CIP (Approval No. 18-04, 18-12, 18-13, and 20-01).

The experiment was carried out according to the Law for the Humane Treatment and Management of Animals Standards Relating to the Care and Management of Laboratory Animals and Relief of Pain (Ministry of the Environment, Japan), the Fundamental Guidelines for Proper Conduct of Animal Experiments and Related Activities in Academic Research Institutions (Ministry of Education, Culture, Sports, Science and Technology, Japan), and the Guidelines for Proper Conduct of Animal Experiments (the Science Council of Japan). Animal health and well-being was also assessed in accordance with the guidelines described above.

At the end of the experiment, whole blood was collected by cardiac puncture under anesthesia with 4% isoflurane vapor. Plasma/serum were collected after centrifugation for 10 min at 500 ×*g* for cytokine analysis and limulus amebocyte lysate (LAL) assay. Peripheral white blood cells were collected for RNA analysis. Bone marrows (BMs) were collected for isolation of CD11b^+^ cells, RNA analysis, and primary culture. Hippocampuses were collected and frozen at -80°C for cytokine analysis. Brains were collected for histopathological analysis and isolation of microglia.

### LPS Treatment

Purified LPS derived from *P. agglomerans* (Macrophi Inc., Kagawa, Japan) was used in this study. LPS derived from *P. agglomerans* was purified to over 99% according to the methods described previously (11). LPS derived from *P. agglomerans* has very low nucleic acid and protein contamination [protein contamination was 0.5%, nucleic acid contamination was less than 0.35% (w/w)] and activates macrophages in very small amounts (1.6 ng/ml) (11). It was confirmed that the LPS content of the D12450B diet was lower than that of the common mouse diet MF (Oriental Yeast Co., Ltd, Tokyo, Japan) or CE-2 (CLEA Japan Inc., Tokyo, Japan) (**Supplementary Table 1**). For oral LPS administration, LPS was dissolved in drinking water (sterile distilled water) and applied at 1 mg/kg body weight (BW)/day. The dose of LPS was determined to be the sufficient dose required to achieve preventive effects as estimated from previous studies (2, 12). The drinking water was changed weekly and the concentration of LPS was adjusted according to the average body weight and amount of water consumption. We previously confirmed that the LPS in drinking water was not significantly degraded in a week (12).

For the DRCD model, LPS was orally administrated to mice 1 week before STZ-injection until the end of the experiment (n = 17–19). For OAL without intracerebroventricular STZ injection, naïve mice were orally administered LPS (1 mg/kg/day, for 1 week) (n = 5). For intraperitoneal administration of LPS, naïve mice were intraperitoneally injected with LPS (4 mg/kg BW, single-dose) (n = 5), and samples were collected 4 h after LPS injection.

### Intracerebroventricular STZ Injection

STZ was purchased from Sigma-Aldrich, St Louis, MO, USA. Under anesthesia with 4% isoflurane vapor, mice were fixed using the brain stereotaxic apparatus SR-5M-HT (Narishige, Tokyo, Japan) and administrated a single injection of STZ (6.6 mg/kg, dissolved in saline 5 µl) into the right lateral ventricle with the microinjector, IMS-20, and micromanipulator SMM-100 (Narishige). The stereotaxic coordinates were + 0.3 mm anterior, + 1.0 mm lateral (right) and + 2.5 mm ventral from bregma. After the skin suture, antibiotic ointment (20 mg/g Chloramphenicol, 5 mg/g Fradiomycin, 100,000 U/g Nystatin, Daiichi Sankyo Healthcare Co., Ltd, Tokyo, Japan) was dabbed on the wound. Control sham mice were administrated 5 µl saline into the right lateral ventricle. After the surgery, mice were monitored daily for pain/discomfort and infections in accordance with the guidelines described above. We checked for proper placement of the needle by delivering 7 μl of 5% Trypan Blue Dye (Nacalai, Kyoto, Japan) (**Supplementary Figure 1**).

### Microglia Depletion

The CSF1R inhibitor PLX3397 (Pexidartinib) was purchased from Chemgood, Glen Allen, VA, USA. PLX3397 was formulated based on the D12450B diet (Research Diets) at a concentration of 400 mg/kg chow. The components of the PLX3397-containing diet are shown in **Supplementary Table 2**. Mice were orally administered PLX3397 and LPS 1 week before STZ-injection until the end of the experiment.

### Morris Water Maze (MWM) Test

To assess spatial learning and memory, the MWM test was carried out 3 weeks after STZ-injection as previously described (2), with minor modifications. Briefly, the experimental apparatus consisted of a circular tank 100 cm in diameter and 40 cm in height filled with water to 30 cm, maintained at 23 ± 1°C and rendered opaque with white ink. The area of the pool was conceptually divided into four equal quadrants, and cards with different shapes (circle, square, triangle or cross) were placed on the wall of each quadrant. A removable circular platform 10 cm in diameter was submerged 1 cm below the water surface and placed approximately in the midpoint of one quadrant, defined as the target quadrant. Each mouse consecutively received a pre-training session (1 day), training session (4 days), and probe session (1 day).

One day before the first training session, each mouse received a pre-training session to make it aware of the escape platform. The mouse was put on the platform for 20 s, given a 30 s free swim, and then assisted in swimming back to the platform. The next day, the first training session was conducted to assess spatial learning ability. From the next day, training session continued for four consecutive days. One each trial, the mouse was released into the water at a randomly assigned starting position, facing the pool’s wall. The mouse was given 60 s to find the platform and was allowed to stay on it for 20 s after reaching the escape platform. The spatial learning ability of each mouse was identified as the time elapsed between releasing and locating the platform, defined as escape latency. If the mouse failed to find the platform within 60 s, it was gently guided to the platform and kept there for 20 s, and the escape latency was recorded as 60 s. Each mouse underwent four trials per day, and the average value of the escape latency was calculated. One day after the last training session, the probe test was performed to assess the spatial reference memory ability of the mice. The platform was removed from the pool, and each mouse was placed in the pool at a starting position located opposite the target quadrant and allowed to swim freely for 60 s. The time spent swimming in the target quadrant was recorded. A video camera was hung above the center of the pool to record the swimming paths of the mice. The swimming trajectory was visualized and the swimming distance/velocity was measured using AnimalTracker software (13).

### Histochemical Analysis

Brains were fixed with 4% paraformaldehyde and embedded in paraffin. The cut surface was unified at the same position of the hippocampal dentate gyrus. Klüver-Barrera staining, Gallyas-Braak staining, and immunostaining of Iba1 were performed with the standard procedure. Briefly, Klüver-Barrera staining was performed as follows. The tissues were stained with 0.1% Luxol fast blue at 56°C overnight and differentiated in 0.05% lithium carbonate solution for 30 s. Then, the tissues were counterstained with 0.1% cresyl violet acetate for 10 min. For Gallyas-Braak staining, the tissues were placed in 5% periodic acid for 5 min and incubated in silver iodide solution for 1 min. After washing in 0.5% acetic acid, the tissues were placed in developer solution for 10–20 min. After washing in 0.5% acetic acid, the tissues were incubated in 0.1% gold chloride for 5 min. Then, the tissues were fixed in 1% sodium thiosulfate for 5 min and counterstained with 0.1% nuclear fast red for 2 min.

For immunostaining of Iba1, the tissues were incubated with anti-Iba1 antibody at room temperature (RT) for 15 min (Wako, Osaka, Japan) after blocking. Then, the tissues were incubated with HRP-conjugated anti-rabbit IgG antibody at 4°C for 1 h (Leica Biosystems, Buffalo Grove, IL, USA). Enzymatic color development was performed using DAB (Leica Biosystems).

To evaluate cerebral atrophy, the thickness of the cerebral cortex was measured using imaging software NIS-Element (Nikon, Tokyo, Japan) in the Klüver-Barrera stained brains. For quantitative analysis of tau inclusion, argyrophilic grains were counted in Gallyas-Braak stained brains.

### Isolation of Primary Microglia From Adult Mouse

Primary microglial cells were isolated from adult mouse brain by enzymatic digestion as described previously (14), with minor modifications. Briefly, the brain was removed and kept in ice cold PBS. The brain tissue was chopped finely with a fine sharp razor. The whole brain homogenate was then incubated in DMEM containing 1.2 units/ml dispase II, 1 mg/ml papain (Sigma-Aldrich), 100 units/ml penicillin and 100 μg/ml streptomycin (Thermo Fisher Scientific, Waltham, MA, USA), 20 units/ml RNase inhibitor (Promega, Madison, WI, USA) and 10 units/ml DNase I (Takara Bio, Shiga, Japan) for 30 min at 37°C. The digestion was terminated by the addition of PBS containing 10% fetal bovine serum (FBS; Invitrogen, Carlsbad, CA, USA), and the cells were then centrifuged for 5 min at 300 ×*g* at RT. The pellets were resuspended in PBS containing 0.5% bovine serum albumin (BSA; Sigma-Aldrich) and 2 mM EDTA (Wako), then centrifuged for 5 min at 300 ×*g* at RT. The cell suspension was passed through a 70-μm cell strainer (Corning, Durham, NC, USA) and myelin was removed using the debris removal solution (Miltenyi Biotec, Bergisch Gladbach, Germany). After myelin removal, the cells were incubated with anti-CD11b antibodies conjugated to magnetic beads (20 μl/brain; Miltenyi Biotec) in PBS containing 0.5% BSA and 2 mM EDTA for 15 min at 4°C. After washing, the CD11b^+^ cells were separated using autoMACS^®^ proseparator (Miltenyi Biotec) and used as microglia. As a control, CD11b^+^ cells were isolated from BM in a similar manner.

### Flow-Cytometric Analysis of Iba1

Isolated CD11b^+^ cells from brain or BM were fixed and permeabilized using fixation/permeabilization solution (BD Biosciences, San Jose, CA, USA) for 20 min at 4°C. After washing, the cells were incubated with anti-Iba1 antibody (Wako) for 30 min at 4°C followed by incubation with Alexa Fluor 555-conjugated anti-rabbit IgG (Thermo Fisher Scientific) for 30 min at 4°C. The median of fluorescent intensity (MFI) was measured in total 5,000-count cells using a Beckman Coulter Gallios flow cytometer and Kaluza for Gallios software version 1.3 (Beckman Coulter, Indianapolis, IN, USA).

### Determination of Aβ_1-42_ and Cytokines (ELISA)

The levels of Aβ_1-42_ in isolated microglia were determined using a commercial ELISA kit (Wako). Protein was extracted from isolated microglia in ice-cold 70% (v/v) formic acid (Wako, Tokyo, Japan) followed by centrifugation at 20,000 ×*g* for 1 h at 4°C. The supernatant was neutralized with a 20-fold dilution in 1M Tris buffer, and used for Aβ_1-42_ ELISA. The concentration of Aβ_1-42_ was normalized against the number of isolated microglia.

The levels of IL-6 and TNF-α in plasma were determined using a commercial ELISA kit (BioLegend, San Diego, CA, USA). The levels of CSF1 in plasma and brain were determined using a commercial ELISA kit (R&D systems, Minneapolis, MN, USA). The snap-frozen brain tissues were homogenized in ice-cold PBS containing 1% protease inhibitor cocktail (GE Healthcare UK Ltd., Buckinghamshire, UK) and 1% phosphatase inhibitor cocktail (Nacalai), followed by sonication for 5 min and centrifugation at 12,000 ×*g* for 10 min at 4°C. The supernatant was used for CSF1 ELISA. The total protein concentration in each sample was determined by a BCA assay kit (Thermo Fisher Scientific) and the concentration of target protein in tissues were reported as pictograms of cytokine relative to the protein content. Absorbance was measured using the iMark microplate reader (BIO RAD, Hercules, CA, USA), and data was analyzed using the Microplate Manager 6 software (BIO RAD).

### Microarray Analysis

Microarray analysis was performed at the Cell Innovator, Fukuoka, Japan. Briefly, the RNA samples of microglia were quantified with Agilent 2200 TapeStation (Agilent Technologies, Santa Clara, CA, USA). Each 50 ng of total RNA was labeled using a Low-Input QuickAmp Labeling Kit (Agilent Technologies). The RNA was hybridized with SurePrint G3 Mouse GE microarray 8 × 60K v2 (Agilent Technologies) using pooled sample (n = 4), and the signals were detected using a DNA microarray scanner (Agilent Technologies). Data was quantified by Feature Extraction software (Agilent Technologies). Normalization was performed by a quantile algorithm with statistical processing software R [R Core Team (2019). *R: A Language and Environment for Statistical Computing.* R Foundation for Statistical Computing, Vienna, Austria].

The ratio (non-log-scaled fold change) and Z scores (15) were calculated from the normalized signal intensities of each probe for comparison between control and experimental samples. Then, the criteria for regulated genes were defined as follows: for upregulated genes, Z score ≧ 2.0 and ratio ≧ 1.5-fold; for downregulated genes, Z score ≦ 2.0 and ratio ≦ 0.66 (16). The heat map was created by using the distance from the median of the log2 converted signal value with MultiExperiment Viewer (MeV) (17, 18).

### Quantitative RT-PCR

RNA was extracted using the RNeasy micro kit (QIAGEN, Hilden, Germany) and cDNA was synthesized using the reverse transcription using ReverTra Ace qPCR RT Master Mix (TOYOBO, Osaka, Japan), according to the manufacturer’s instructions. Real-time PCR assay was carried out using 2 μl of cDNA as the template and 10 μl of Power SYBR Green PCR Master Mix (Thermo Fisher Scientific) on the Stratagene Mx 3005P QPCR System (Agilent Technologies, Santa Clara, CA, USA).

The primers are listed in **Supplementary Table 3**. *m-CSF1* and *s-CSF1* were distinguished by specific primers (19). The data was analyzed based on 2^−ΔΔCt^ method and normalized by GADPH expression using the MxPro software version 4.10 (Agilent Technologies). The thermal cycling conditions for the PCR were 95°C for 10 min for polymerase activation, followed by 45 cycles of 95°C for 15 s for denaturation, and 60°C for 1 min for extension.

### LAL Assay

The total abundance of LPS in serum was analyzed as described previously (2) using a kinetic turbidimetric assay with the Limulus Amebocyte Lysate (LAL) assay kit and a Toxinometer ET-6000 computer operated kinetic incubating tube reader (Wako). The serum was diluted 1:10 in pyrogen-free water (Otsuka Pharmaceutical Factory, Inc., Tokushima, Japan) and preheated to 70°C for 10 min prior to analysis. Data was analyzed using the Toximaster QC7 software.

### Cell Culture

Bone marrows were collected from femurs by flushing with PBS. After RBC lysis, BMC (1 × 10^7^ cells/ml) were seeded in 96-well tissue culture plates (n = 5) and cultured in Dulbecco’s modified Eagle’s medium (Wako, Osaka, Japan) supplemented with 10% fetal bovine serum (Sigma-Aldrich, St Louis, MO, USA), 100 U/ml penicillin and 100 μg/ml streptomycin (Life Technologies, Carlsbad, CA, USA), at 37°C in 5% CO_2_. To evaluate CSF1 production by LPS, BMC were treated with or without LPS (1–10^4^ pg/ml, purified LPS derived from *Pantoea agglomerans*, Macrophi Inc., Kagawa, Japan). RNA and culture supernatants were collected at 4 h and 24 h after the last LPS treatment, respectively.

The murine microglial cell line C8-B4 microglia, which was established from brain cerebella, was purchased from the American Type Culture Collection, Manassas, VA, USA. C8-B4 microglia (2 × 10^5^ cells/ml) were seeded in 12-well tissue culture plates (n = 3, in triplicate) and cultured in Dulbecco’s modified Eagle’s medium (Wako) supplemented with 10% fetal bovine serum (Sigma-Aldrich), 100 U/ml penicillin and 100 μg/ml streptomycin (Life Technologies), at 37°C in 5% CO_2_. C8-B4 microglia were treated with or without 80 ng/ml CSF1 (Promega). After 16 h of incubation, the culture supernatant was collected and RNA was extracted from the C8-B4 microglia.

For the phagocytosis assay, C8-B4 microglia were incubated with HiLyte Fluor 488-labeled Aβ_1-42_ (1 μg/ml; AnaSpec, Fremont, CA, USA) or fluorescent latex beads (Fluoresbrite YG Microspheres 2.0 μm; Polysciences, Warrington, PA, USA) at a cell/bead ratio of 1:5 for 3 h after 16 h of treatment with CSF1. After washing with PBS, C8-B4 microglia were detached by 0.25% trypsin treatment (Life Technologies), and the MFI of phagocytosed beads in the cells were measured using a Beckman Coulter Gallios flow cytometer with Kaluza software (Beckman Coulter).

To evaluate the neuroprotective effect of MCS, a murine neuroblast cell line Neuro-2a, which was established from brain neuroblastoma, was cultured with CSF1-treated MCS. Neuro-2a cells were provided from the Japanese Collection of Research Bioresources Cell Bank, Osaka, Japan. Neuro-2a cells were seeded in 24-well tissue culture plates (4 × 10^5^ cells/ml) and pre-cultured in Eagle’s minimal essential medium with non-essential amino acids (Wako) supplemented with 10% fetal bovine serum (Sigma-Aldrich), 100 U/ml penicillin and 100 μg/ml streptomycin (Life Technologies), at 37°C in 5% CO_2_ for 24 h (n = 3). Then, the medium was replaced by CSF1-treated MCS. After culturing with CSF-1 treated MCS for 24 h, 400 µM STZ (Sigma-Aldrich) was added to the culture medium, and Neuro-2a cells were cultured for another 24 h. Finally, the cell number was counted after detachment by treatment with 0.25% trypsin for 2 min, and the survival rate was calculated by staining with Trypan Blue Dye (Nacalai).

### Statistical Analysis

Statistical analysis was performed using the GraphPad Prism 6.0 software package (GraphPad Software Inc., San Diego, CA). Results were presented as the mean ± standard error of the mean (SE). The differences between the groups of mice were analyzed by one-way ANOVA followed by Tukey’s multiple comparisons test or two-way ANOVA followed by Sidak multiple comparisons test. Student’s t test was used to compare differences from two independent groups. A *p*-value < 0.05 was considered significantly different. Representative experiments were conducted at least twice, independently.

## Results

### Prevention of DRCD by OAL

**Figure 1:**
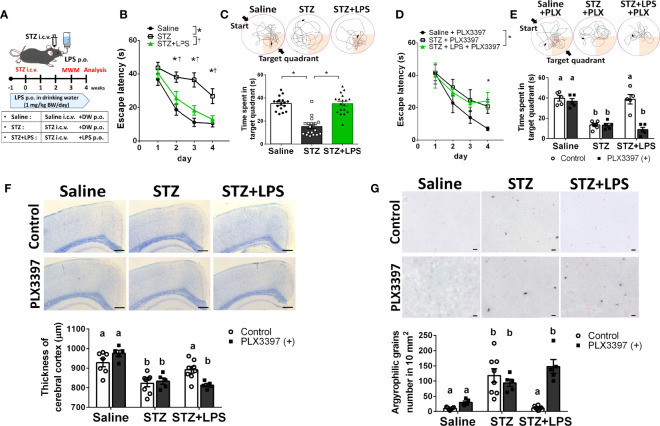
Microglia are indispensable for preventing diabetes-related cognitive dysfunction (DRCD) through oral administration of LPS (OAL). **(A)** The experimental schedule of DRCD and OAL. **(B, C)** Prevention of deterioration of the learning ability and memory performance induced by OAL (n = 17–19). **(D, E)** PLX3397 reversed the prevention of DRCD by OAL (n = 5–7). **(F)** Prevention of cerebral cortex atrophy by OAL (n = 5–8). Bars, 200 μm. **(G)** Prevention of accumulation of argyrophilic grains by OAL (n = 5–8). Bars, 10 μm. Means ± SE are presented. * ^,†^ *p* or *^a,b^p* < 0.05 for one-way ANOVA with Tukey’s multiple comparisons or two-way ANOVA with Sidak multiple comparisons. DW, distilled water; i.c.v., intracerebroventricular; p.o., per os.

To demonstrate DRCD prevention through OAL, spatial learning memory was evaluated using the Morris water maze (MWM) procedure with the STZ-induced DRCD model (**Figure 1A**). In training session, STZ-injected mice showed reduced learning ability as their escape latency was significantly longer than saline-injected controls and LPS-treated mice. By contrast, the loss of learning ability was prevented by OAL. The escape latency of LPS-treated mice was comparable to saline-injected controls (**Figure 1B**). In probe test, the time spent in the target quadrant of STZ-injected mice was significantly shorter than saline-injected controls, indicating the declined memory performance. On the contrary, LPS-treated mice did not decline memory ability as they exhibited significantly longer time spent in the target quadrant than STZ-injected mice (**Figure 1C**). As shown in **Figure 1C**, it was evident from the swimming trajectory that LPS-treated mice memorized the target location. Furthermore, the athletic ability such as velocity and total swimming distance did not significantly vary between the groups (**Supplementary Figure 2**). Therefore, these results indicate that OAL prevents DRCD.

### Microglia Are Indispensable for Preventing DRCD Through OAL

To investigate whether microglia are necessary for DRCD prevention through OAL, we depleted microglia using the PLX3397 (20, 21). Microglia depletion by PLX3397 was confirmed by reduced number of cells expressing Iba1 (**Supplementary Figure 3**). It was demonstrated that microglia depletion reversed the preventive effect of OAL on DRCD (**Figures 1D, E**). Furthermore, microglial depletion did not reduce the spatial learning memory of saline-injected healthy controls

Next, to investigate the pathological mechanism of DRCD prevention through OAL, a histological analysis of the brain was performed. STZ injection induced cerebral cortex atrophy, whereas OAL prevented diabetes-induced cerebral atrophy, which was ablated by microglial depletion (**Figure 1F**). As shown in **Figure 1G**, Gallyas-Braak staining revealed diabetes-induced accumulation of argyrophilic grains, which are mainly composed of hyperphosphorylated tau protein (22). Contrarily, the argyrophilic grains were hardly observed in the brains of saline-injected controls and LPS-treated mice. The prevention of argyrophilic grain accumulation by OAL was cancelled by PLX3397 administration. These results indicate that suppression of cerebral atrophy and tau phosphorylation *via* microglia are the preventive mechanisms of DRCD through OAL.

### OAL-Microglia Are Distinct From Inflammatory Microglia Induced by Systemic Injection of LPS

**Figure 2:**
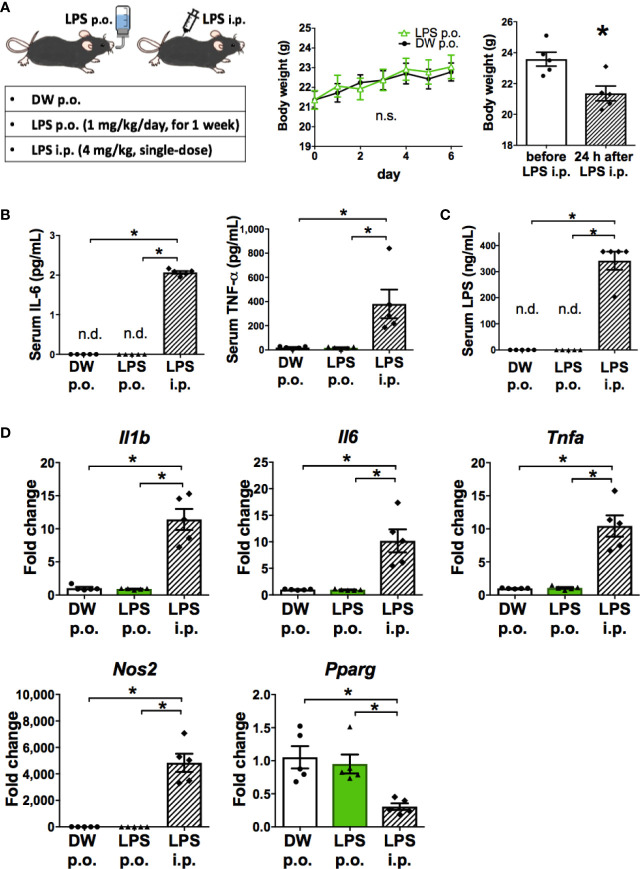
OAL-microglia are distinct from inflammatory microglia transformed by intraperitoneal injection of LPS. **(A)** No weight loss by OAL in naïve mice without STZ i.c.v. (n = 5). **(B)** No increase in serum inflammatory cytokines by OAL. **(C)** Undetectable serum LPS by OAL. **(D)** OAL-microglia showed distinct gene expression patterns from inflammatory microglia transformed by LPS i.p. Means ± SE are presented. *p < 0.05 for Student’s t test, one-way ANOVA with Tukey’s multiple comparisons, or two-way ANOVA with Sidak multiple comparisons. n.s., not significant; n.d., not detected; i.p., intraperitoneal.

The characteristics of microglia transformed by OAL (OAL-microglia) in naïve mice were compared to that of inflammatory microglia induced by intraperitoneal injection with LPS (LPS i.p.). First, the systemic effects of LPS administration were evaluated. As shown in **Figures 2A, B**, OAL did not induce weight loss and systemic elevation of inflammatory cytokines, unlike LPS i.p. (4 mg/kg). In addition, LPS was not detected in the blood of mice that received OAL unlike LPS i.p. (**Figure 2C**).

Second, gene expression analysis of OAL-microglia in naïve mice showed that OAL-microglia did not promote inflammatory mediators (IL-1β, IL-6, TNF-α, and NOS2) nor suppress anti-inflammatory mediator (PPARγ), unlike microglia of mice that received LPS i.p. (**Figure 2D**). These results indicate that OAL-microglia are distinct from inflammatory microglia transformed by LPS i.p. These results indicate that the characteristics of OAL-microglia are fundamentally different from those of inflammatory microglia induced by systemic LPS injection.

### Characterization of Neuroprotective Microglia Transformed Through OAL

**Figure 3:**
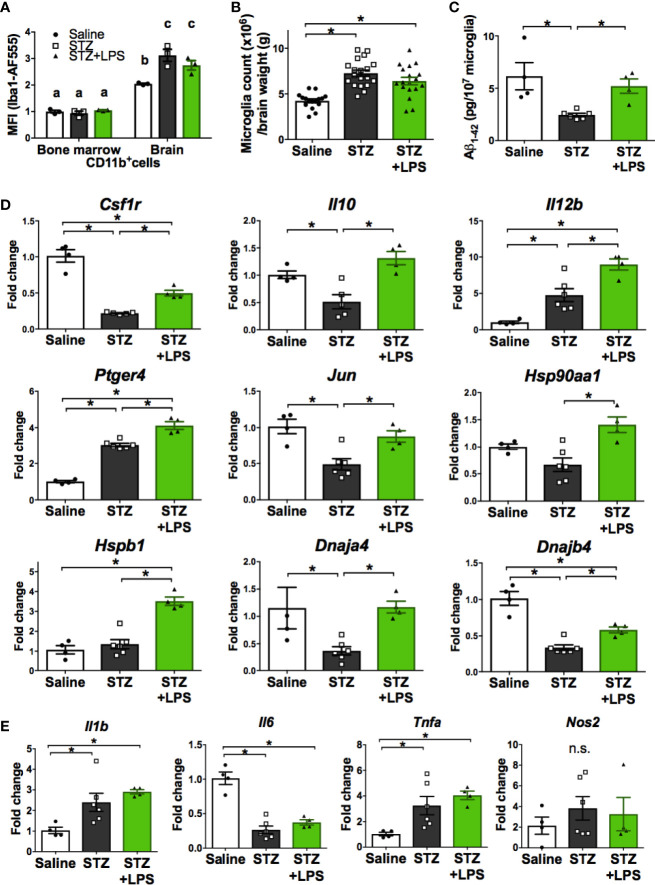
Neuroprotective microglia are transformed through OAL. **(A)** High expression of Iba1 in isolated microglia (n = 3). **(B)** Number of isolated microglia (n = 17–19). **(C)** Aβ_1-42_ levels in isolated microglia (n = 4–6). **(D, E)** Upregulation of neuroprotective genes and unchanged inflammatory genes in isolated microglia through OAL (n = 4–6). Means ± SE are presented. *p or *^a,b^p* < 0.05 for one-way ANOVA with Tukey’s multiple comparisons or two-way ANOVA with Sidak multiple comparisons. n.s., not significant. ^a,b,c^p < 0.05 for two-way ANOVA with Sidak multiple comparisons. The different letters (the symbols of a, b, c) indicate statistically significant difference between groups in multiple comparison test.

We next performed further characterization of OAL-microglia in the DRCD model. First, it was confirmed that isolated microglia, not peripheral monocytes, highly express Iba1 (microglia marker), indicating that the contamination of peripheral monocytes was extremely low (**Figure 3A**). In addition, the number of microglia was not significantly different between STZ-injected and LPS-treated mice (**Figure 3B**).

As shown in **Figure 3C**, Aβ contained in isolated microglia was declined in STZ-injected mice, but was not declined in LPS-treated mice as well as in saline-injected controls. whereas the diabetes-induced suppression of Aβ phagocytosis by microglia was prevented by OAL. The result indicates that OAL promotes Aβ phagocytosis of microglia.

Second, comprehensive gene analysis by microarray was used to extract the notable differentially expressed genes from the overall tendency of gene expression. Microarray analysis using pooled sample provided 1,061 candidate genes upregulated in OAL-microglia (**Supplementary Material 1** and **Supplementary Figure 4A**), showing that the gene expression patterns of OAL-microglia are distinct from healthy microglia and microglia in DRCD-developed mice (DRCD-associated microglia). Additionally, gene ontology term analysis revealed that pathways related to immune function, neuronal survival, and tissue repair were upregulated in OAL-microglia (**Supplementary Figure 4B**).

Quantitative RT-PCR was performed to distinguish representative neuroprotective genes from those fluctuating in OAL-microglia. As shown in **Figure 3D**, the following neuroprotective genes are significantly upregulated in OAL-microglia compared to DRCD-associated microglia: CSF1R, IL-10, IL-12B, prostaglandin-E_2_ EP4 receptor (encoded by *Ptger4* gene), c-Jun, and heat shock protein (HSP) family (encoded by *Hsp90aa1*, *Hspb1*, *Dnaja4*, and *Dnajb4* genes). In addition, gene expression of IL-12B, EP4 receptor, and HSPβ1 was promoted in OAL-microglia compared to microglia of healthy mice. However, OAL did not affect the gene expression of inflammatory mediators in microglia. The gene expression of inflammatory mediators, such as IL-1β, IL-6, tumor necrosis factor (TNF)-α and nitric oxide synthase (NOS) 2, was not significantly different between OAL-microglia and DRCD-associated microglia (**Figure 3E**). These results indicate that OAL-microglia, which are characterized by high expression of neuroprotective genes, differ in gene expression from DRCD-associated microglia and microglia of healthy mice.

### CSF1R Signaling Is Involved in the Transformation to Neuroprotective Microglia Through OAL

**Figure 4:**
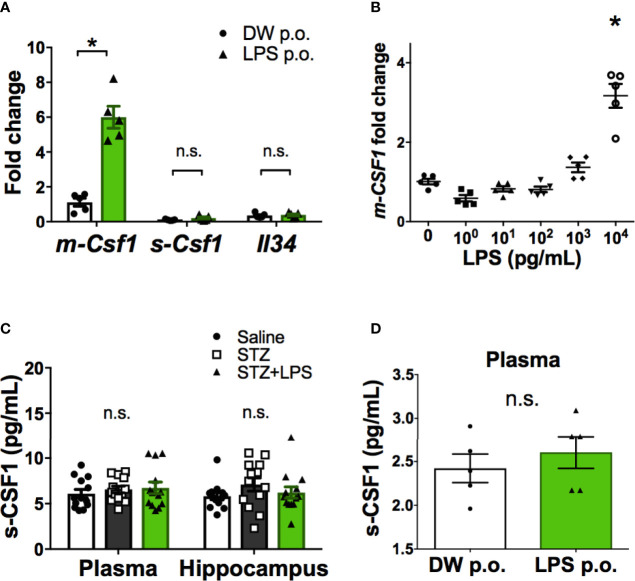
Promotion of m-CSF1 on peripheral blood leukocytes by OAL. **(A)** Upregulated *m-Csf1* through OAL in peripheral blood leukocytes (n = 5). **(B)** Upregulated *m-Csf1* by LPS in primary bone marrow cells. **(C, D)** No increase in s-CSF1 levels through OAL in DRCD [**(C)**, n = 13] and naïve mice [**(D)**, n = 5]. Means ± SE are presented. *p < 0.05 for Student’s t test or one-way ANOVA with Tukey’s multiple comparisons. n.s., not significant.

Ligands for CSF1R include CSF1 and IL-34. CSF1 is also expressed as two isoforms, the membrane-bound form (m-CSF1) and secretory form (s-CSF1) (23). Gene expression analysis of CSF1R ligands in peripheral leukocytes revealed that only m-CSF1 expression was promoted by OAL (**Figure 4A**). LPS stimulation promoted m-CSF1 gene expression in primary cultured bone marrow cells (**Figure 4B**). However, OAL did not alter the expression of s-CSF1 and IL-34 in peripheral leukocytes (**Figures 4A, C, D**). These results suggested that CSF1R activation by m-CSF1 of peripheral leukocytes induces transformation to neuroprotective OAL-microglia.

**Figure 5:**
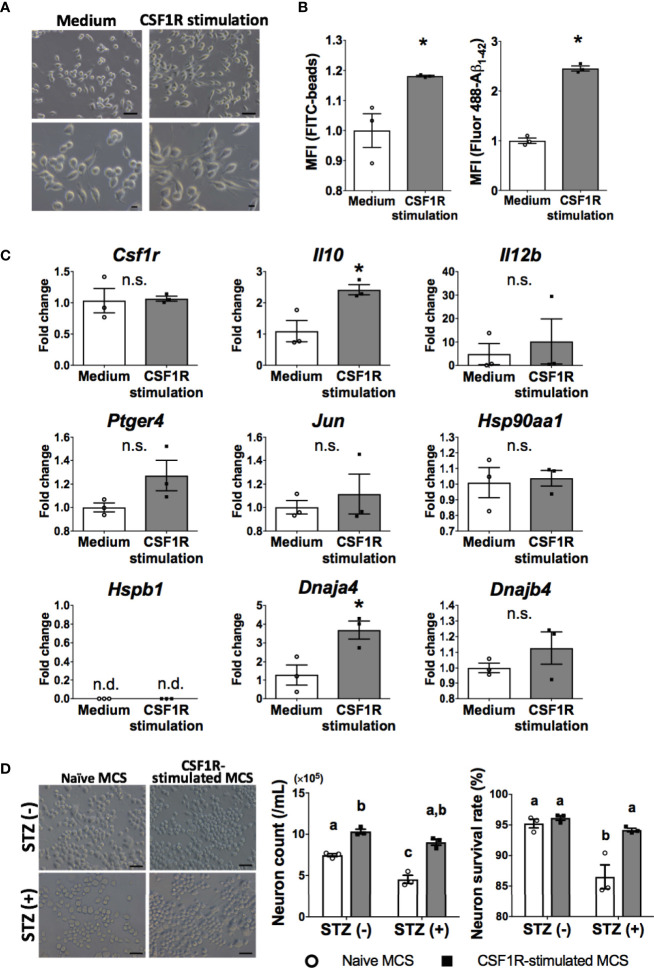
Neuroprotective microglia are transformed by CSF1R signaling. **(A)** Dendrite protrusion of CSF1R-stimulated microglia. Bars, 50 (upper) and 10 μm (lower). **(B)** Promoted phagocytosis of CSF1R-stimulated microglia. **(C)** Upregulation of neuroprotective genes in CSF1R-stimulated microglia. **(D)** Prevention of diabetes-induced neuronal death by CSF1R-stimulated MCS. Bar, 50 μm. Means ± SE are presented. *p or *^a,b^p* < 0.05 for Student’s t test or two-way ANOVA with Sidak multiple comparisons. MCS, microglia culture supernatant. n.s., not significant; n.d., not detected. ^a,b,c^p < 0.05 for two-way ANOVA with Sidak multiple comparisons. The different letters (the symbols of a, b, c) indicate statistically significant difference between groups in multiple comparison test.

Thus, we examined whether neuroprotective transformations similar to OAL-microglia *in vivo* could be reproduced through CSF1R-stimulation of microglia *in vitro*. C8-B4 microglia, one of the most widely used *in vitro* assay systems, is a cell line whose homology with microglia *in vivo* has been sufficiently confirmed based on marker expression and immune responsiveness (**Figure 5A** and **Supplementary Figure 5**) (24–27). Therefore, the cell line was used to characterize CSF1R-stimulated microglia *in vitro*.

Consistent with OAL-microglia *in vivo*, CSF1R-stimulated microglia showed promotion of phagocytosis (**Figure 5B**). In addition, CSF1R-stimulated microglia also promoted gene expression of IL-10 and HSP40 (encoded by *Dnaja4* gene) (**Figure 5C**).

To investigate whether CSF1R-stimulated microglia prevent diabetes-induced neuropathy similar to OAL-microglia *in vivo*, STZ was applied to neurons treated with CSF1R-stimulated microglia culture supernatant (CSF1R-stimulated MCS). As shown in **Figure 5D**, it was demonstrated that CSF1-treated MCS suppressed STZ-induced neuronal cell death (**Figure 5D**). indicating the neuroprotective effect of CSF1R-stimulated microglia. These results demonstrate that neuroprotective transformations like OAL-microglia *in vivo* can be reproduced with CSF1R-stimulated microglia.

## Discussion

Our previous studies have revealed that OAL is effective in maintaining homeostasis (2, 12). We previously showed that the immunoprotective effect of OAL increases depending on the LPS dose. Phagocytosis of peritoneal macrophages induced by OAL is enhanced with higher LPS dose (10~1000 μg/kg/day) (28). In high-fat diet-fed apoE-deficient mice, the preventive effect of OAL to suppress weight gain and increasing blood glucose level is observed at higher LPS doses (1 mg > 0.3 mg/kg/day) (12). Similarly, in senescence-accelerated prone 8 mice fed a high-fat diet, the preventing effect of OAL on cognitive dysfunction is found at higher LPS doses (1 mg > 0.3 mg/kg/day) (2). Based on the above results, the LPS dose was set to 1 mg/kg/day in this study.

Here, we demonstrate the effect of OAL on preventing DRCD as MMM test showed that OAL prevented the loss of both learning (**Figure 1B**) and memory ability (**Figure 1C**) induced by diabetes. The preventing effect of OAL on high fat diet-induced cognitive dysfunction has also been demonstrated in our previous study, in which mice were individually bred to accurately control LPS intake (2). Because the preventing effect of OAL on cognitive dysfunction in this study is consistent with the previous study, it is considered that there is almost no variation in the amount of water consumed among individuals. In addition, we previously reported that the phagocytic activity-enhancing effect of peritoneal macrophages induced by OAL was not observed in Toll-like receptor 4 (TLR4)-deficient mice (28). Therefore, the protective effects induced by OAL are considered to be TLR4 dependent. Furthermore, considering that the LPS derived from *P. agglomerans* used in this study has very low nucleic acid and protein contamination and activates macrophages in very small amounts (1.6 ng/ml) (11), it is reasonable to conclude that the effect obtained is due to LPS derived from *P. agglomerans*.

Microglia play a central role in the innate immune system and the maintenance of homeostasis in the central nervous system (CNS). Since our previous study suggested that OAL enhances phagocytosis of amyloid beta (Aβ) by microglia (2), we considered that microglia may also play an important role in the prevention of DRCD through OAL. According to our hypothesis, microglia depletion using PLX3397 revealed that microglia are necessary for DRCD prevention through OAL (**Figures 1D, E**).

Furthermore, microglial depletion did not reduce the spatial learning memory of saline-injected healthy controls, which was consistent with a previous study (20). Because PLX3397 does not affect the number of other CNS cell types and circulating leukocyte subsets (20, 21), these results indicate that microglia are indispensable for the prevention of DRCD through OAL, although they do not affect cognitive ability under physiological conditions.

Histological analysis showed that OAL prevented diabetes-induced cerebral atrophy and the accumulation of argyrophilic grains (**Figures 1F, G**), which are mainly composed of hyperphosphorylated tau protein and are frequently seen in association with neurodegeneration and cognitive decline (22). Therefore, it is indicated that suppression of cerebral atrophy and tau phosphorylation *via* microglia are the preventive mechanisms of DRCD through OAL.

Thus, while microglia are necessary to prevent DRCD through OAL, DRCD cannot be prevented without OAL, even in the presence of microglia. It was shown that the characteristics of OAL-microglia are distinct from those of DRCD-associated microglia. Therefore, transformation to the neuroprotective microglia induced by OAL is important for DRCD prevention.

Based on the above results, we next characterized the OAL-microglia. Generally, LPS is known to induce inflammation through transformation into inflammatory microglia when systemically injected (29, 30). Therefore, the characteristics of OAL-microglia in naïve mice were compared to that of inflammatory microglia.

As shown in **Figure 2**, unlike LPS i.p., OAL did not induce side effects such as weight loss and systemic inflammation (**Figures 2A, B**), or inflammatory microglia (**Figure 2D**). It has been reported that immune training (immune tolerance) with repetitive low-dose LPS i.p. transforms microglia into a neuroprotective phenotype. However, mild sickness, weight loss, and systemic inflammation are often associated with LPS i.p. (31). In contrast, our results challenge the stereotype of LPS as an inflammation inducer by showing that the characteristics of OAL-microglia are fundamentally different from those of inflammatory microglia induced by systemic LPS injection. Additionally, LPS was not detected in the blood of mice that received OAL unlike LPS i.p. (**Figure 2C**), indicating that orally administrated LPS does not directly impact microglia.

Furthermore, it was revealed that characteristics of OAL-microglia were distinct form DRCD-associated microglia (**Figure 3**). Because the number of microglia was not significantly different between STZ-injected and LPS-treated mice (**Figure 3B**), we concluded that OAL induces qualitative rather than quantitative transformation of microglia. Consistent with our previous studies which suggested that OAL-microglia contributed to the prevention of cognitive dysfunction by promoting the phagocytosis of Aβ (2, 28), the diabetes-induced suppression of Aβ phagocytosis by microglia was prevented by OAL (**Figure 3C**). This is considered a background mechanism for preventing tau phosphorylation by OAL (**Figure 1G**) since Aβ deposition triggers tau protein phosphorylation leading to neuropathy (32).

Gene analysis revealed that some neuroprotective genes are highly expressed in OAL-microglia compared to DRCD-associated microglia (**Figure 3D**). CSF1R regulates survival and proliferation of macrophages and its signal induces transformation into neuroprotective microglia (33–36). IL-10 is an anti-inflammatory cytokine and interestingly synergizes with IL-12B to regulate inflammation in tumor models (37). Activation of EP4 receptor suppresses brain inflammation (38–40), and also synergizes with CSF1 signaling (41). c-Jun forms activator protein 1 (AP-1), a transcription factor, which is activated downstream of CSF1 signal involving in transformation to anti-inflammatory macrophage (34, 42). AP-1 is also involved in the induction of IL-10, IL-12, HSP (43–46). *Hsp90aa1*, *Hspb1*, *Dnaja4*, and *Dnajb4* genes encode HSP family, and exogenous HSP promotes Aβ phagocytosis of microglia and induces neuroprotection (47). These results indicate that OAL-microglia are neuroprotective and qualitatively different from DRCD-associated microglia.

Considering our results above, we speculated that there must be a mediator that connects OAL and the transformation to neuroprotective microglia in the DRCD model. We focused on CSF1R as a potential mediator as it was one of the highly expressed genes in OAL-microglia. It has been reported that CSF1R signaling induces transformation to neuroprotective microglia *via* c-Jun (33–36, 42).

Furthermore, peripheral levels of CSF1, a CSF1R ligand, correlate with CSF1R levels in the CNS (48, 49), and peripheral administration of CSF1 improves neurological disorders (50–52). Therefore, we hypothesized that OAL increases expression of the peripheral CSF1R ligand, which activates the CSF1R signal to induce the transformation to neuroprotective microglia.

It was revealed that gene expression of m-CSF1, not s-CSF1 or IL-34, was promoted by OAL in peripheral leukocytes (**Figures 4A, C, D**), and that LPS stimulation promoted m-CSF1 gene expression in primary cultured bone marrow cells (**Figure 4B**). Therefore, it is suggested that m-CSF1, whose expression is promoted on peripheral leukocytes by OAL, is a mediator that induces transformation to neuroprotective OAL-microglia *via* CSF1R. It is yet unsolved mechanisms by which OAL promotes only m-CSF1, not s-CSF1. Concerning the point above, since *Csf1* gene is transcribed into m-CSF1 or s-CSF1 mRNA forms *via* alternative splicing (23), OAL is thought to influence the regulation of mRNA splicing of *Csf1* gene.

Since both m-CSF1 and s-CSF1 bind to CSF1R and induce transformation to homogeneous macrophages (53), the main difference between the two is assumed to be the mechanism of transmission. In other words, s-CSF1 acts systemically in an endocrine manner, whereas m-CSF1 acts locally in a juxtacrine or paracrine manner (23). Since CSF1R is specifically expressed in microglia in the brain, it is considered that m-CSF1, whose expression is promoted on peripheral leukocytes by OAL, may activate CSF1R signaling in microglia *via* the “just-in-time system” method.

Schwartz et al. reported that the cerebral choroid plexus functions as a location for crosstalk between CNS cells and the peripheral immune system (54). Under physiological conditions, peripheral immune cells regularly patrol to maintain CNS homeostasis in the choroid plexus. In contrast, under pathological conditions, peripheral immune cells sense the abnormality in the choroid plexus and prevent neuropathy through crosstalk with CNS cells by secreting neuroprotective molecules. Therefore, the cerebral choroid plexus is likely to act as a communication site for restrictive signaling between m-CSF1 of peripheral leukocytes and CSF1R on microglia.

In agreement with the theory of Schwartz et al., OAL had little effect on the expression of neuroprotective genes in the microglia of naïve mice (**Supplementary Figure 6**). Therefore, it is considered that CSF1R on microglia is activated by m-CSF1 of peripheral leukocyte only under pathological conditions such as CNS diabetes, which induces transformation to neuroprotective microglia to prevent DRCD. Assuming such an abnormality detection system, m-CSF1 is more plausible than s-CSF1.

Finally, we investigated whether CSF1R stimulation could reproduce the characteristics of OAL-microglia (**Figure 5**). It was shown that CSF1R-stimulated microglia promoted phagocytosis (**Figure 5B**) and gene expression of IL-10 and HSP40 (encoded by *Dnaja4* gene) (**Figure 5C**). Since the expression of the EP4 receptor and other HSPs is promoted downstream of IL-10 (55, 56), it can be said that CSF1R-stimulated microglia almost reproduces the characteristics of OAL-microglia *in vivo*, such as high phagocytosis and neuroprotective gene expression. Furthermore, since CSF1R-stimulated microglia prevented diabetes-induced neuronal death (**Figure 5D**), it is indicated that OAL-microglia *in vivo* can be reproduced with CSF1R-stimulated microglia. Therefore, it is strongly proposed that transformation to neuroprotective microglia *via* CSF1R activation is involved in the mechanism of DRCD prevention by OAL.

To prove our hypothesis that CSF1R signal mediates the transformation to neuroprotective OAL-microglia *in vivo*, we needed a system that functionally inhibits only the CSF1R signal without affecting the survival of microglia. However, the problem is that inhibition of CSF1R induces microglia death because the CSF1-CSF1R signal controls not only the function of microglia but also their survival and proliferation (20, 21). In fact, PLX3397 used for microglia depletion in this study is a CSF1R inhibitor. Therefore, the next task is to develop alternative approaches to test our hypothesis. Our discovery that the CSF1R activation by m-CSF1 is a key factor that mediates the transformation to neuroprotective OAL-microglia is a major step towards elucidating the DRCD prevention mechanism through OAL.

**Figure 6:**
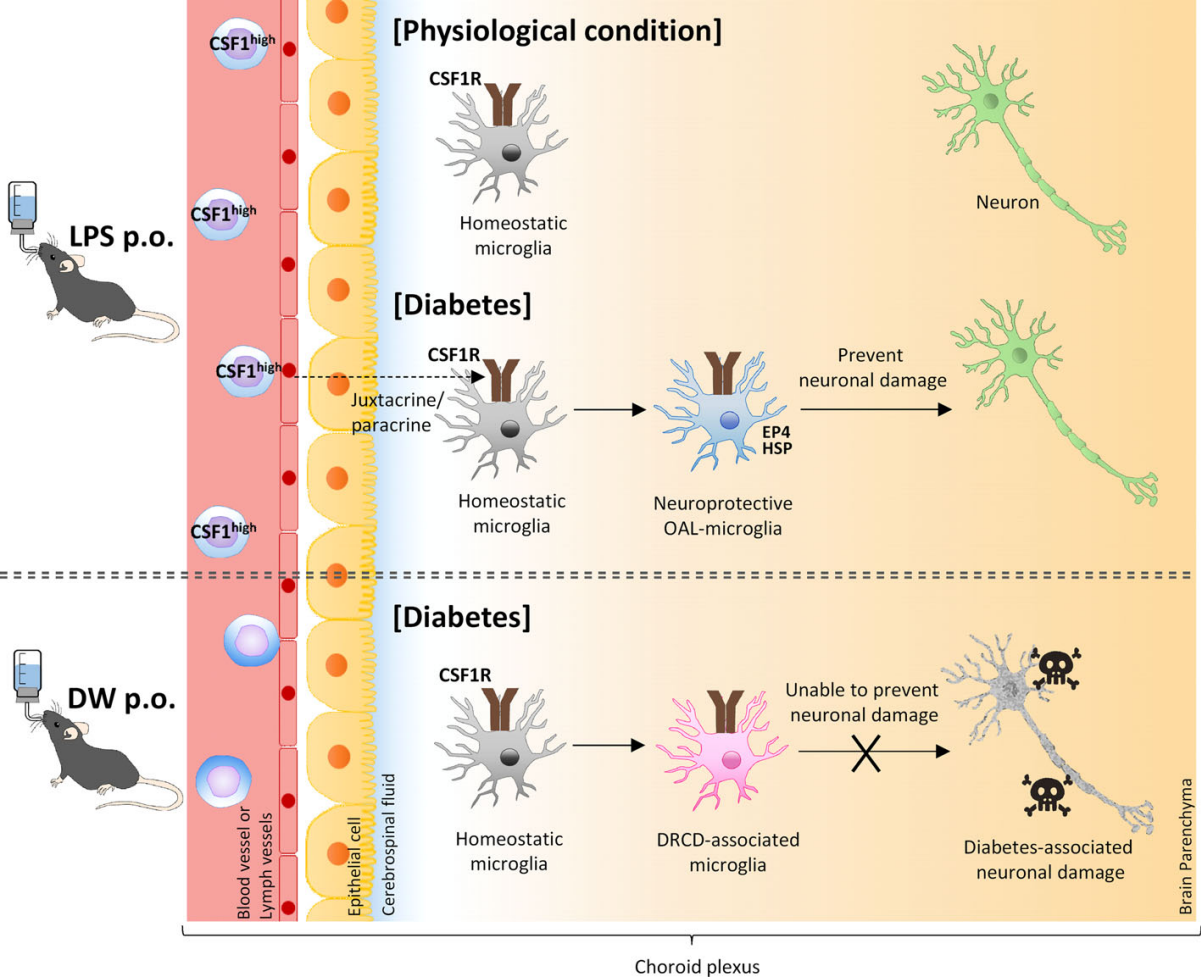
A model of transformation into neural microglia through OAL. OAL induces m-CSF1 expressing leukocytes, which patrol and maintain CNS homeostasis under physiological conditions. Under diabetes-induced pathological conditions, microglia transform to a neuroprotective phenotype mediated by m-CSF1 in a juxtacrine/paracrine manner, and prevent neuronal damage.

In conclusion, the present study demonstrates that OAL prevents DRCD by transforming microglia to a neuroprotective phenotype. In addition, activation of CSF1R on microglia mediated by m-CSF1 expressed on peripheral leukocytes strongly suggests that the CSFR1-mCSF1 interaction facilitates the mechanism of microglia transformation (**Figure 6**). Other groups also have reported that physiological mucosal exposure to LPS maintains homeostasis *via* tissue-resident macrophages (57, 58), which supports our findings.

Microglia depletion has been recently proposed as a new solution to neuropathy. Indeed, microglia shift to a pro-inflammatory phenotype in some neurological diseases, and therefore microglia depletion leads to anti-inflammatory and neuroprotective effects (21, 59). However, since microglia play diverse roles in CNS homeostasis, microglia depletion is an impractical strategy leading to collapse of the brain immune system and homeostasis (60, 61). However, it is an innovative method of preventing DRCD by controlling microglial transformation in a simple and safe way through OAL.

Previous study compared the biological activity of LPS from *P. agglomerans* with that of LPS from *Escherichia coli* and that of LPS from *Enterobacterium* (the rice symbiotic bacterium) at the cellular level, showing that there is not much difference in the biological activity of the three (62). However, LPS derived from *P. agglomerans* has been proven safe in animal studies when administered orally (no-observed adverse-effect level 4500 mg/kg/day) (5–7). Also in humans, it is confirmed that 10 μg/kg/day OAL does not induce systemic inflammation by measuring biomarkers such as white blood cell count, red blood cell count, aspartate aminotransferase, alanine aminotransferase, creatinine, C-reactive protein, and immunoglobulin A in human peripheral blood samples (63). Moreover, OAL at the dose improves hyperglycemia, hyperlipidemia (64), reduced bone density (65), and blood flow (63). In addition, because LPS from *P. agglomerans* is abundant in the organic foods we consume on a daily basis (6, 66–68), humans have a dietary experience of LPS from *P. agglomerans*. Therefore, although more careful safety studies will be required in the future, LPS from *P. agglomerans* is thought to be the suitable LPS considering the application of OAL to human therapy.

Based on the hygiene hypothesis (69), human beings used to intake LPS naturally from the organic environment. Accordingly, microglia used to be activated physiologically (6, 66, 67). However, the present age is described as the LPS-lost era, in which human beings live in an unnaturally clean and inorganic environment. In a sense, OAL is like a time machine that returns microglia back to the good old LPS-abundant era, as if *DeLorean* of “*Back to the Future*”.

## Data Availability Statement

The original contributions presented in the study are included in the article/**Supplementary Material**. Further inquiries can be directed to the corresponding author.

## Ethics Statement

The animal study was reviewed and approved by the Animal Care and Use Committee of the Control of Innate Immunity CIP.

## Author Contributions

HM, HI, and G-IS conceptualized the study and coordinated the experiments. HM, KY, and MY performed the experiments. HM performed data curation and formal analysis. YN and CK provided resource of LPS. HI and G-IS acquired the funding and administrated the project. HM wrote the manuscript supervised by G-IS, HI, CK, and YN with contribution from all authors. All authors contributed to the article and approved the submitted version.

## Funding

This study was funded by the Control of Innate Immunity Collaborative Innovation Partnership. This study was also supported by a grant from the Cross-ministerial Strategic Innovation Promotion Program (SIP-No. 14533073) of the Council for Science from Technology and Innovation (CSTI) in the Cabinet Office of the Japanese Government and the National Agriculture and Food Research Organization (NARO). CSTI and NARO had no role in the study design, data collection and analysis, decision to publish, or preparation of the manuscript.

## Conflict of Interest

HM, KY, MY, HI, and G-IS are employed by the Control of Innate Immunity, Collaborative Innovation Partnership. YN, HI, CK, and G-IS are employed by Macrophi Inc. This does not affect our adherence to journal policies on sharing data and materials.

## Publisher’s Note

All claims expressed in this article are solely those of the authors and do not necessarily represent those of their affiliated organizations, or those of the publisher, the editors and the reviewers. Any product that may be evaluated in this article, or claim that may be made by its manufacturer, is not guaranteed or endorsed by the publisher.
