# Supplementary material for: Prevention of Diabetes-Associated Cognitive Dysfunction Through Oral Administration of Lipopolysaccharide Derived From Pantoea agglomerans
Source: Front Immunol. 2021 Aug 27;12:650176. doi: 10.3389/fimmu.2021.650176 (PMC8429836; doi:10.3389/fimmu.2021.650176)
Supplement: Supplementary file 1 [file DataSheet_1.pdf]

Prevention of diabetes-associated cognitive dysfunction through oral administration of lipopolysaccharide derived from *Pantoea agglomerans*

Haruka Mizobuchi[1*], Kazushi Yamamoto[1], Masashi Yamashita[1], Yoko Nakata[2], Hiroyuki Inagawa[1,2,3], Chie Kohchi[1,2] & Gen-Ichiro Soma[1,2,3]

[1] *Control of Innate Immunity, Collaborative Innovation Partnership, Kagawa, Japan*

[2] *Macrophi Inc., Kagawa, Japan*

[3] *Research Institute for Healthy Living, Niigata University of Pharmacy and Applied Life Sciences, Niigata, Japan*

*Corresponding Author: Haruka Mizobuchi

Address: 2217-16 Hayashi-cho, Takamatsu-shi, Kagawa 761-0301, Japan

Phone: +81-87-813-9201

Fax: +81-87-813-9203

Email address: mizobuchi@shizenmeneki.org

ORCID: 0000-0002-6667-4820

SUPPLEMENTARY INFORMATION

Supplementary Tables

Supplementary Table 1 | LPS concentration in mouse diets.

Diets	LPS in diets (ng/g)	Estimated daily intake (µg/head)
MF (Oriental Yeast Co., Ltd)	760.3	2-4
CE-2 (CLEA Japan, Inc)	1,322.5	3-5
D12450B (Research Diets)	4.2	<0.02

Supplementary Table 2 | The components of the PLX3397-containing diet.

**Product**	**D12450B control diet**		**D12450B with 400 mg PLX3397/kg diet**	
%	*gm*	*kcal*	*gm*	*kcal*
Protein	19	20	19	20
Carbohydrate	67	70	67	70
Fat	4	10	4	10
Total		100		100
Kcal/gm	3.8		3.8	
Ingredient	*gm*	*kcal*	*gm*	*kcal*
Casein	200	800	200	800
L-Cystine	3	12	3	12
Corn Starch	315	1,260	315	1,260
Maltodextrin 10	35	140	35	140
Sucrose	350	1,400	350	1,400
Cellulose	50	0	50	0
Soybean Oil	25	225	25	225
Lard	20	180	20	180
Mineral Mix S10026	10	0	10	0
DiCalcium Phosphate	13	0	13	0
Calcium Carbonate	5.5	0	5.5	0
Potassium Citrate, 1 H2O	16.5	0	16.5	0
Vitamin Mix V10001	10	40	10	40
Choline Bitartrate	2	0	2	0
PLX3397 (99% purity)	0	0	0.426	0
FD&C Yellow Dye #5	0.05	0	0	0
**Total**	**1,055.05**	**4,057**	**1,055.426**	**4,057**
**PLX3397 (mg/kg diet)**	**0**		**400**	

Supplementary Table 3 | List of primers used for quantitative RT-PCR.

Gene	Forward	Reverse
m-Csf1	5′-CAACTCAGCCACCCCCGTT-3′	5′-TCCTCCTT CAGCTCTGCG-3′
s-Csf1	5′-CCAAGAACTGCAACAACAGCTTTG-3′	5′-AGTCAGCAAGACTAGGATGATGC-3′
Csf1r	5′-CAGTTCAGAGTGATGTGTGGTC-3′	5′-CTTGTTGTTCACTAGGATGCCG-3′
Dnaja4	5′-GACGAGGCCAGGATCTGA-3′	5′-TCATCACCCCCTTCTCCA-3′
Dnajb4	5′-AAAGAGGTCGCAGAAGCGTAT-3′	5′-TCTCCGTGGAAAGTGTACCTG-3′
Gapdh	5′-CGACTTCAACAGCAACTCCCACTCTTCC-3	5′-TGGGTGGTCCAGGGTTTCTTACTCCTT-3′
Hsp90aa1	5′-CGGACGCTCTGGATAAAATCC-3′	5′-TCCTTCCCCGAGTCCAGTTT-3′
Hspb1	5′-TGCTTCACCCGGAAATACAC-3′	5′-CTCGAAAGTAACCGGAATGG-3′
Il10	5′-GCTGGACAACATACTGCTAACC-3′	5′-CCCAAGTAACCCTTAAAGTCCTG-3′
Il12b	5′-ACAGCACCAGCTTCTTCATCAG-3′	5′-TCTTCAAAGGCTTCATCTGCAA-3′
Il1b	5′-GAAAGACGGCACACCCACCCT-3′	5′-GCTCTGCTTGTGAGGTGCTGATGTA-3′
Il34	5′-ACTCAGAGTGGCCAACATCACAAG-3′	5′-ATTGAGACTCACCAAGACCCACAG-3′
Il6	5′-CCAGAGATACAAAGAAATGATGG-3′	5′-ACTCCAGAAGACCAGAGGAAAT-3′
Jun	5′-TGGGCACATCACCACTACAC-3′	5′-TCTGGCTATGCAGTTCAGCC-3′
Nos2	5′-GTTCTCAGCCCAACAATACAAGA-3′	5′-GTGGACGGGTCGATGTCAC-3′
Pparg	5′-CCATTCTGGCCCACCAAC-3′	5′-AATGCGAGTGGTCTTCCATCA-3′
Ptger4	5′-ATGGTCATCTTACTCATCGCCAC-3′	5′-CTTTCACCACGTTTGGCTGAT-3′
Tnfa	5′CTGTGAAGGGAATGGGTGTT-3′	5′-GGTCACTGTCCCAGCATCTT-3′

Supplementary Figures

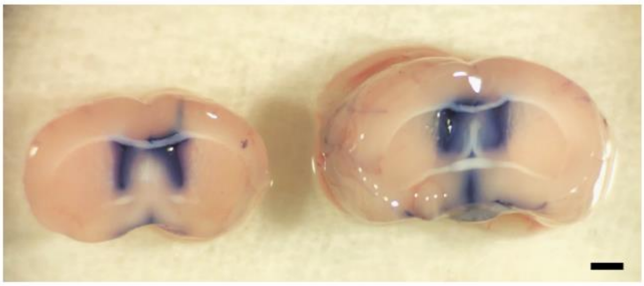

Supplementary Figure 1 | Intracerebroventricular administration of Trypan blue. Intracerebroventicular injection was confirmed by delivering 7 µl of 5% Trypan blue Dye (Nacalai, Kyoto, Japan). The photograph shows a representative image of the coronal plane at the bregma of the brain 15 min after dye administration. It was confirmed that the ventricles were stained blue. Bar, 1 mm.

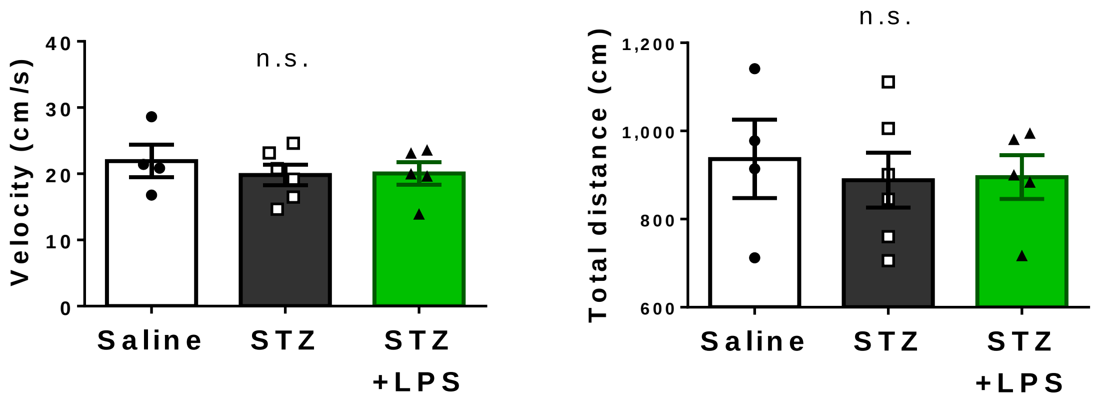

Supplementary Figure 2 | Velocity and total swim distance of MWM. No difference in velocity and total swim distance between the groups in MWM (n = 4–6). Mean ± SE of each group are presented. *$p < 0.05$ for one-way ANOVA with Tukey's multiple comparisons test. n.s., not significant.

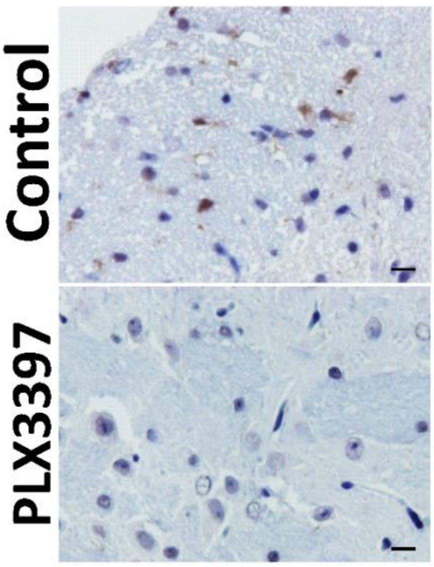

Supplementary Figure 3 | Reduction of Iba1-expressing cells in the brain through PLX3397 administration. Cells stained brown are Iba1-expressing cells. It was confirmed that almost no Iba1-expressing cells were observed in the brain of PLX3397-administered mice. Bars, 10 µm.

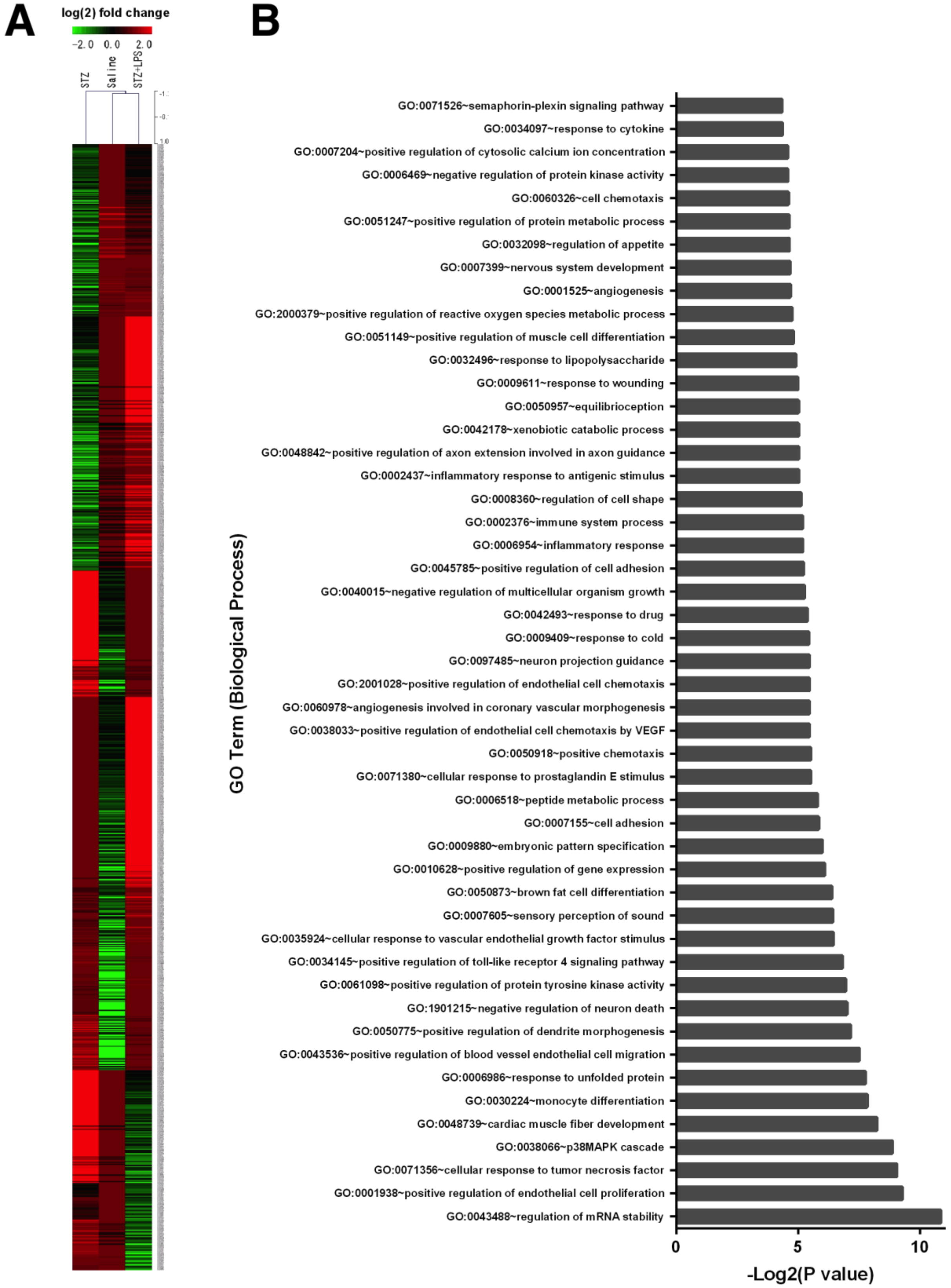

Supplementary Figure 4 | Alteration of the gene expression profile of microglia through OAL.

The gene expression of microglia was comprehensively analyzed by SurePrint G3 Mouse GE

microarray 8 × 60K v2 (Agilent, Santa Clara, CA, USA) using pooled sample (n = 4). (A) Hierarchy clustering analysis of 1,061 genes whose expression was altered in microglia through OAL. Hierarchy clustering analysis was performed using MultiExperiment Viewer (MeV) (70,71). (B) Gene ontology (GO) term analysis of genes whose expression was changed in microglia through OAL. GO term analysis was performed using DAVID Bioinformatics Resources (72,73).

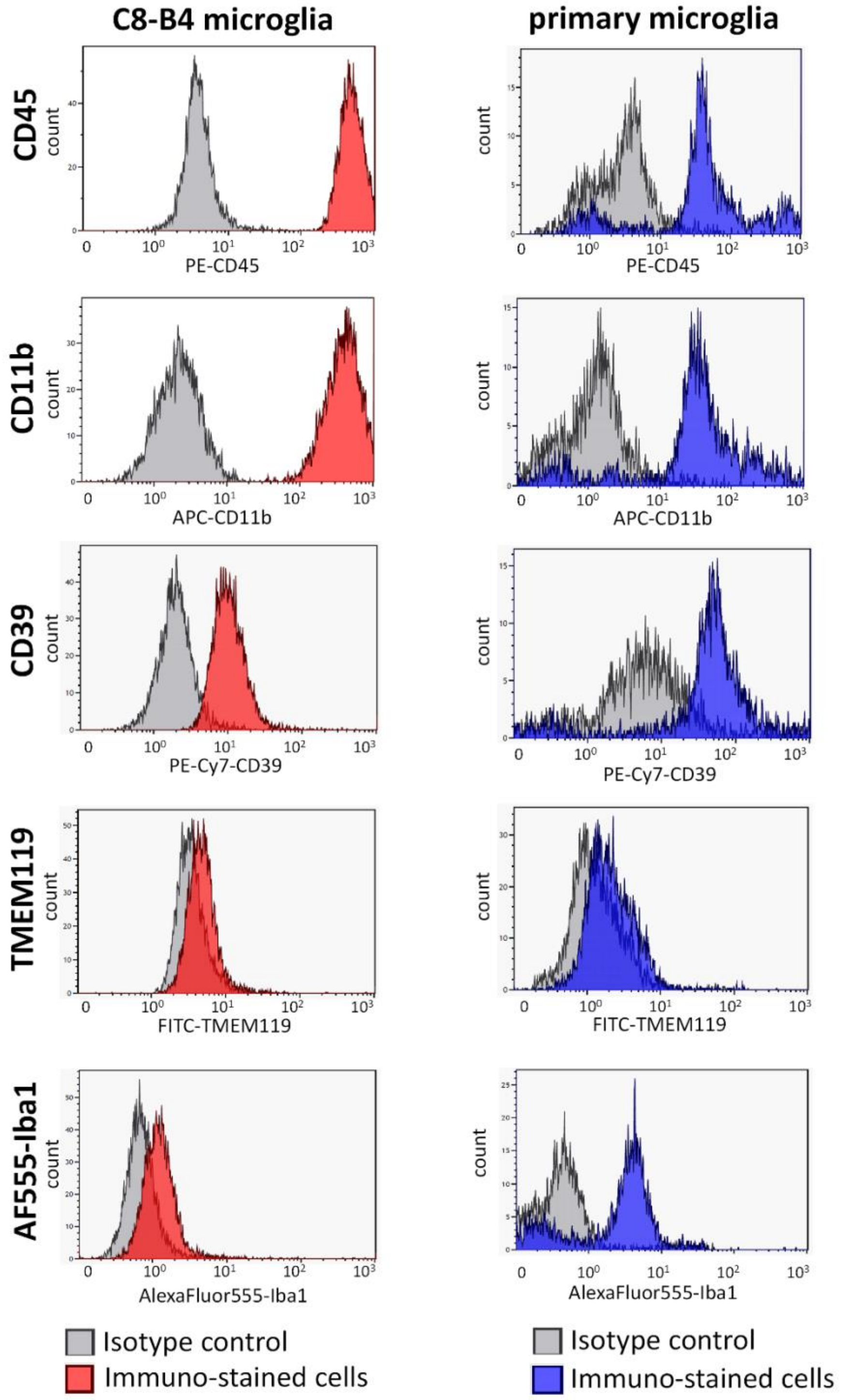

Supplementary Figure 5 | Similarity of the characteristics between the C8-B4 microglia cell line and primary microglia. Both the C8-B4 microglia cell line and primary microglia were characterized by the microglia markers CD11b, CD45, CD39, TMEM119, and Iba1. C8-B4 microglia cell line and

primary microglia were stained with APC- labeled anti-CD11b antibody (BioLegend), PE- labeled anti-CD45 antibody (BioLegend), PE-Cy7-labeled anti-CD39 antibody (BioLegend), FITC-labeled anti-TMEM119 antibody (Abcam), or anti-Iba1 antibody (Wako) followed by AlexaFluor 555-labeled anti-rabbit IgG (Thermo Fisher Scientific). Representative histograms are shown. A total of 5,000-cells were assessed using a Beckman Coulter Gallios flow cytometer and Kaluza software. Representative histograms are shown.

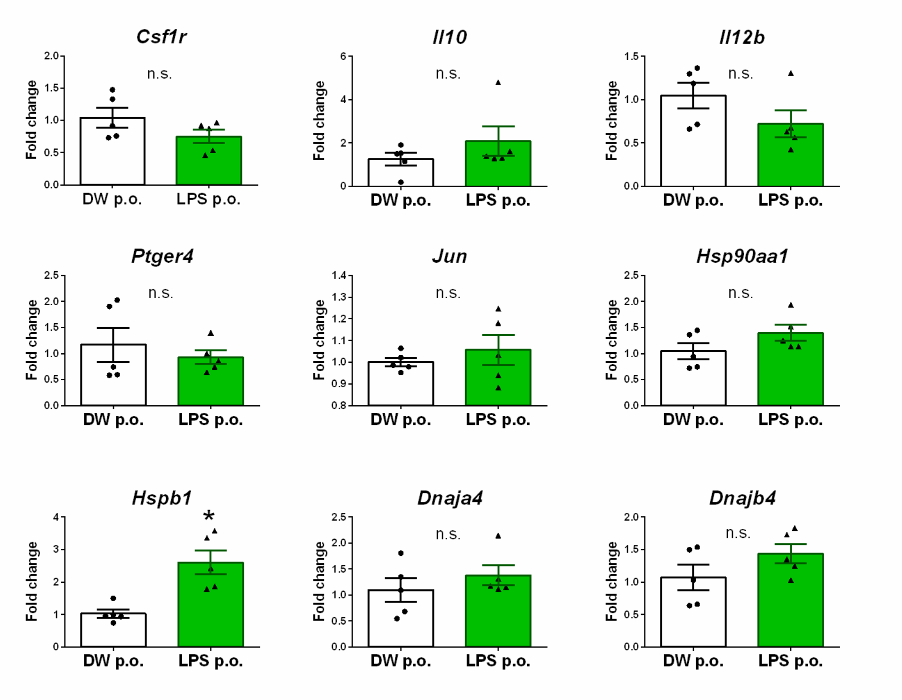

Supplementary Figure 6 | Upregulation of neuroprotective gene expression through OAL was not induced in the microglia of naïve mice without STZ injection. Mean ± SE of each group are presented. *$p < 0.05$ for Student's t-test. DW, distilled water; n.s., not significant; p.o., per os.

REFERENCES

70. Saeed AI, Sharov V, White J, Li J, Liang W, Bhagabati N, Braisted J, Klapa M, Currier T, Thiagarajan M, et al. TM4: A free, open-source system for microarray data management and analysis. *Biotechniques* (2003) **34**:374–378. doi:10.2144/03342mt01

71. Saeed AI, Bhagabati NK, Braisted JC, Liang W, Sharov V, Howe EA, Li J, Thiagarajan M, White JA, Quackenbush J. TM4 Microarray Software Suite. *Methods Enzymol* (2006) **411**:134–193. doi: 10.1016/S0076-6879(06)11009-5

72. Huang DW, Sherman BT, Lempicki RA. Systematic and Integrative Analysis of Large Gene Lists Using DAVID Bioinformatics Resources. *Nat Protoc* (2009) **4**:44–57. doi: 10.1038/nprot.2008.211

73. Huang DW, Sherman BT, Lempicki RA. Bioinformatics enrichment tools: Paths toward the comprehensive functional analysis of large gene lists. *Nucleic Acids Res* (2009) **37**:1–13. doi:10.1093/nar/gkn923
